# *miR-185-5p* regulates the proliferation and differentiation of neural stem/progenitor cells

**DOI:** 10.3389/fcell.2024.1510746

**Published:** 2024-12-05

**Authors:** Xuanran Feng, Xue Du, Xiaoyu Yang, Changqi Chen, Zhanping Liang, Xiaonan Xu, Yi Wang, Jialin C. Zheng, Xiaohuan Xia, Jianhui Liu

**Affiliations:** ^1^ Department of Anesthesiology, Tongji Hospital, School of Medicine, Tongji University, Shanghai, China; ^2^ Translational Research Center, Shanghai Yangzhi Rehabilitation Hospital, School of Medicine, Tongji University, Shanghai, China; ^3^ State Key Laboratory of Cardiovascular Diseases and Medical Innovation Center, Shanghai East Hospital, School of Medicine, Tongji University, Shanghai, China; ^4^ Center for Translational Neurodegeneration and Regenerative Therapy, Tongji Hospital affiliated to Tongji University School of Medicine, Shanghai, China; ^5^ Shanghai Frontiers Science Center of Nanocatalytic Medicine, Tongji University, Shanghai, China; ^6^ Translational Research Institute of Brain and Brain-Like Intelligence, Shanghai Fourth People’s Hospital affiliated to Tongji University School of Medicine, Shanghai, China; ^7^ Collaborative Innovation Center for Brain Science, Tongji University, Shanghai, China

**Keywords:** miR-185-5p, neural stem/progenitor cells, proliferation, differentiation, astrocytes

## Abstract

**Background:**

MicroRNAs (miRNAs) have emerged as an essential regulator of the cell fate commitment of neural stem/progenitor cells (NPCs), although the impacts of certain miRNAs on NPCs remain vague. The aim of this study is to investigate the regulatory effects of *miR-185-5p* on the cell fate commitment of NPCs.

**Methods:**

We investigated the impact of *miR-185-5p* on the proliferation and differentiation capacities of primary NPCs by manipulating the expression of *miR-185-5p* using specific mimics and inhibitors. The effects of *miR-185-5p* on NPCs was confirmed *in vivo* through stereotactic injection of *miR-185-5p* antagonists to the brains of mice at postnatal day 1 (P1).

**Results:**

The expression levels of *miR-185-5p* kept increasing in the differentiation process of NPCs *in vivo* and *in vitro*. Perturbation of *miR-185-5p*’s function showed that *miR-185-5p* inhibited NPCs’ proliferation and promoted embryonic NPCs to differentiate more favorably to the glial lineage. We then validated the anti-proliferation and pro-glial roles of *miR-185-5p* using NPCs isolated from P1 mouse brains. *In vivo* study further showed enlarged NPCs pools and inhibited gliogenesis in the brains of P1 mice after animals received antagomir-185-5p.

**Conclusion:**

Our study suggests *miR-185-5p* as an important regulator for the proliferation and glial fate commitment of NPCs.

## Introduction

During the development of the vertebrate central nervous system (CNS), multipotent neural stem/progenitor cells (NPCs) give rise to various types of neurons and glial cells in a spatially and temporally conserved pattern (Okano and Temple, 2009). Emerging evidence from multiple approaches suggests that the maintenance and differentiation of NPCs are regulated by dynamic interplay among transcription factors, epigenetic factors, non-coding RNAs (ncRNAs), and cell-extrinsic signals from the microenvironment in which NPCs reside. Among them, the involvement of ncRNAs, particular miRNAs, in NPCs regulation has received increasing attentions in the past decade (Ayana et al., 2017; D'Anca et al., 2022; Sharma et al., 2024; Wakabayashi et al., 2014). Among these ncRNAs, miRNAs are the mostly investigated one.

MicroRNAs (miRNAs) are evolutionary conserved ncRNAs that were first reported in 1993 (Lee et al., 1993; Blakaj and Lin, 2008). Being the smallest type of ncRNAs, miRNAs are typically 22–24 nucleotides in length. They bind to partially complementary sequences in the 3′ untranslated region (3′UTR) of target transcripts and regulate their expression at post-transcriptional level (Carthew and Sontheimer, 2009). miRNAs have the ability to play important roles in stem cell fate commitment, therefore controlling the developmental timing and regeneration capacity of the CNS (Sempere et al., 2004; Coolen and Bally-Cuif, 2009; Lopez-Ramirez and Nicoli, 2014; Ghibaudi et al., 2017). Notably, different miRNAs exhibit diverse impacts on cell fate commitment of NPCs, for instance, members of *let-7* and *miR-106b* miRNA families regulate the proliferation of NPCs (Rybak et al., 2008; Xia et al., 2019a; Xia and Ahmad, 2016), while *miR-9*, *miR-21*, and *miR-124* are important for neuronal induction (Zhao et al., 2009; Visvanathan et al., 2007; Xia et al., 2019b; Ma et al., 2019; Yuan et al., 2021). Our previous study has identified *miR-185-5p* as one differentially expressed miRNA during the differentiation process of retinal stem/progenitor cells (RPCs), NPC-like cells in the retina (Xia et al., 2019b). However, the expression patterns and functions of *miR-185-5p* in NPCs during brain development remain vague (Liu et al., 2019; Değerli et al., 2020). Our study found that the expression of *miR-185-5p* increased with embryonic development, suggesting that *miR-185-5p* may be involved in the regulation of cellular processes in NPCs. Therefore, it is essential to explore new functions of *miR-185-5p* to reveal new factors in the regulation of neural development.

In this study, we found that the expression levels of *miR-185-5p* in mouse brains increased from embryonic stage to adult one. The upregulation of *miR-185-5p* expression was observed in NPCs differentiation process *in vitro*, suggesting the involvement of *miR-185-5p* in neurogenesis. We then investigated the effect of *miR-185-5p* on mouse embryonic NPCs. Our *in vitro* studies showed that *miR-185-5p* is critical for inhibiting the proliferation of NPCs and promoting the differentiation of NPCs. We further verified that *miR-185-5p* is a key regulator in controlling the balance of NPCs proliferation and differentiation by administering antagomir-185-5p *in vivo*. Thus, our observations suggest *miR-185-5p* as a promotor of NPCs differentiation, regardless of the neuronal or glial lineage, shifting fate of NPCs from maintenance to differentiation.

## Methods

### Isolation and enrichment of NPCs

NPCs were isolated from mouse fetal brain tissue as described previously (Ma et al., 2019). Briefly, cortical tissue was isolated from embryonic day 14 (E14) C57BL/6J mice and dissociated into single cells. Single cells were cultured for 3–4 days to form neurospheres in NPCs proliferation medium, which contained NeuroCult^®^ NSC Basal Medium (Stem Cell Technologies), NeuroCult^®^ NSC Proliferation Supplements (Stem Cell Technologies), 20 ng/mL bFGF (BioWalkersville), 20 ng/mL EGF (BioWalkersville), 2 μg/mL heparin (Sigma), N2 supplement, 2 mM l-glutamine, 100 U/mL penicillin, and 100 μg/mL streptomycin. Primary neurospheres were collected, centrifuged at low speed to remove individual cells, dissociated into single cells by accutase (Sigma) at 37°C for 5 min, and a second round of neurospheres was formed in suspension culture. Enriched NPCs were harvested after three rounds of neurosphere formation for NPCs proliferation experiments.

### Differentiation of NPCs

Differentiation of NPCs was performed as described previously (Ma et al., 2019). Briefly, NPCs were seeded on Matrigel-coated coverslips in 24-well plates, 2 × 10^5^ per well, which were supplemented with DMEM/F12 (Gibco) and supplemented with 1 × N2 supplement (Gibco), 1 × B27 supplement (ThermoFisher), 1.0 mM GlutaMAX (ThermoFisher), 10 ng/mL brain-derived neurotrophic factor (BDNF) (Peprotech), 10 ng/mL glial cell line-derived neurotrophic factor (GDNF) (Peprotech), 100  U/mL penicillin, and 100 μg/mL streptomycin. The medium was changed every 3 days.

Astrocyte differentiation medium consisted of DMEM/F12 (Gibco) and supplemented with 1 × N2 supplement (Gibco), 10% FBS (Gibco), 1.0 mM GlutaMAX (ThermoFisher), 100  U/mL penicillin, and 100 μg/mL streptomycin.

### miRNA mimics/inhibitor, and transfection

The mimic negtive control, *miR-185-5p* mimic, inhibitor negtive control, and *miR-185-5p* inhibitor were purchased from GenePharma (GenePharma Co., Ltd., Shanghai). Transfection was performed using Lipofectamine 2000 Reagent (Invitrogen) according to the manufacturer’s instructions.

### miRNA antagomir and administration

The antagomir negative control and antagomiR-185-5p were purchased from GenePharma (GenePharma Co., Ltd., Shanghai). Mice were administered 2 μL of 100 μM antagomirs (antagomir-negative control or antagomiR-185-5p) bilaterally in the lateral ventricles.

### Quantitative real-time polymerase chain reaction (RT-qPCR)

The miRNA and mRNA were isolated from tissue or cell samples using the RNeasy Mini kit (Qiagen) according to the manufacturer’s instructions. Genomic DNA was removed and cDNA was synthesized using the DNase I Digestion Kit (Qiagen) and miScript II Reverse Transcription Kit (Qiagen), respectively. Transcripts were amplified using gene-specific primers (Supplementary Table S1) and SYBR Green PCR kit (Qiagen) with ABI7500 (Applied Biosystems). All RT-qPCR results were measured in triplicate for each sample, and negative controls were used without template blanks. Amplification curves and gene expression were normalized to the housekeeping genes Gapdh (mRNA) and U6 snRNA (miRNA).

### Immunofluorescence staining

For cellular experiments, samples were fixed in 4% formaldehyde for 20 min at room temperature and then washed 3 times with PBS. For animal experiments, mouse brain tissue was cryosectioned and the brain slices were washed 3 times with PBS. Cell samples or brain slices were permeabilized with 0.4% Triton X-100 in PBS for 15 min, blocked with 5% BSA and 10% goat serum in PBS for 1 h at room temperature, and then incubated with primary antibody at 4°C overnight. Samples were washed 3 times with PBS and incubated with secondary antibody for 1 h at room temperature, blocked, and then images were acquired.

### Statistical analyses

All results are shown as the means of at least three independent experiments ± S.D. The statistical difference between two independent groups was analyzed with the unpaired Student’s t-test. Data from multiple groups were statistically assessed by one-way ANOVA followed by Tukey *post hoc* tests. Significance was considered when p < 0.05.

## Results

### *miR-185-5p* is highly expressed during the differentiation of NPCs

**FIGURE 1:**
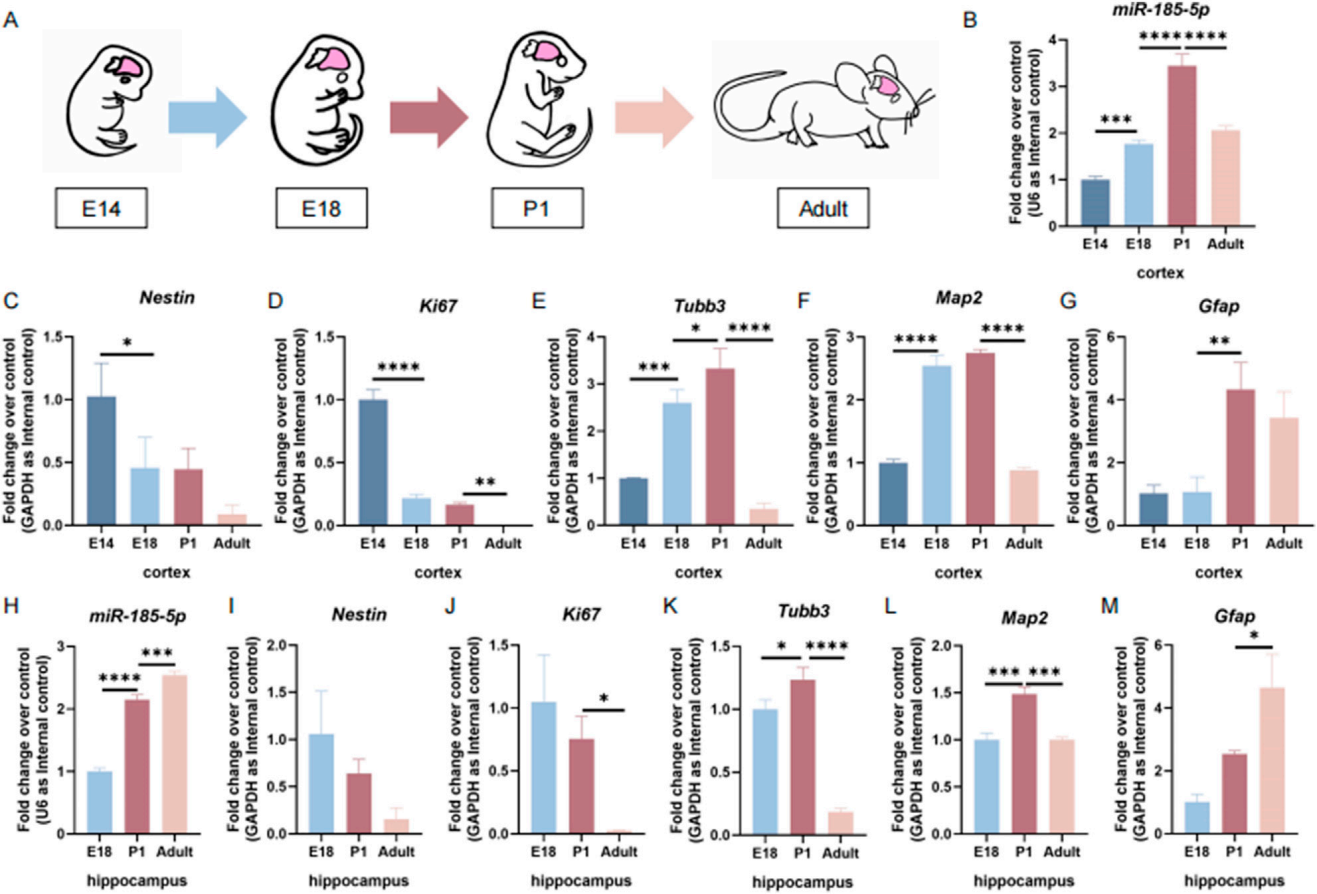
The temporal expression pattern of *miR-185-5p* corresponds to NPCs differentiation *in vivo*. **(A)** A schematic representation of the sample collection during brain development. **(B)** The temporal expression levels of *miR-185-5p* in cortical tissues were determined by RT-qPCR assay. **(C–G)** The expression levels of *Nestin/Ki67/Tubb3/Map2/Gfap* transcripts in cortical tissues were determined by RT-qPCR analysis. **(H)** The temporal expression levels of *miR-185-5p* in hippocampal tissues were determined by RT-qPCR assay. **(I–M)** The expression levels of *Nestin/Ki67/Tubb3/Map2/Gfap* transcripts in hippocampal tissues was determined by RT-qPCR analysis, respectively. Data are mean ± S.D. ∗∗∗∗p < 0.0001, ∗∗∗p < 0.001, ∗∗p < 0.01, and ∗p < 0.05. Experiments were carried out three times in triplicates.

To determine the temporal expression patterns of *miR-185-5p* during the differentiation process of NPCs, we first examined *miR-185-5p* expression levels in the cortical tissues of embryonic day 14 (E14), embryonic day 18 (E18), postnatal day 1 (P1), and adult mice (Figure 1A). We found that the expression levels of *miR-185-5p* increased with brain development from E14 and reached the maximum levels at P1 (Figure 1B). The expression level of *miR-185-5p* in the cortical tissues of adult mice was reduced compared to the P1 stage (Figure 1B). This trend showed a negative correlation with the mRNA levels of *Nestin* (Figure 1C), the expressed transcript corresponding to NPCs, and *Ki67* (Figure 1D), the proliferation marker, and showed a positive correlation with the mRNA levels of the neuronal markers’ transcripts *Tubb3* and *Map2* (Figures 1E, F), and the astrocyte marker *Gfap* (Figure 1G). We further examined the expression level of *miR-185-5p* in the hippocampus and found that the expression levels of *miR-185-5p* in the mouse hippocampi gradually increased from E18 to adulthood (Figure 1H). This trend was opposite to that of *Nestin* (Figure 1I) and *Ki67* (Figure 1J), but not completely consistent with that of *Tubb3* (Figure 1K) and *Map2* (Figure 1L). We found that the expression trend of *miR-185-5p* in the hippocampus was the same as *Gfap* (Figure 1M). The above results suggest that *miR-185-5p* may be related to the differentiation of NPCs.

**FIGURE 2:**
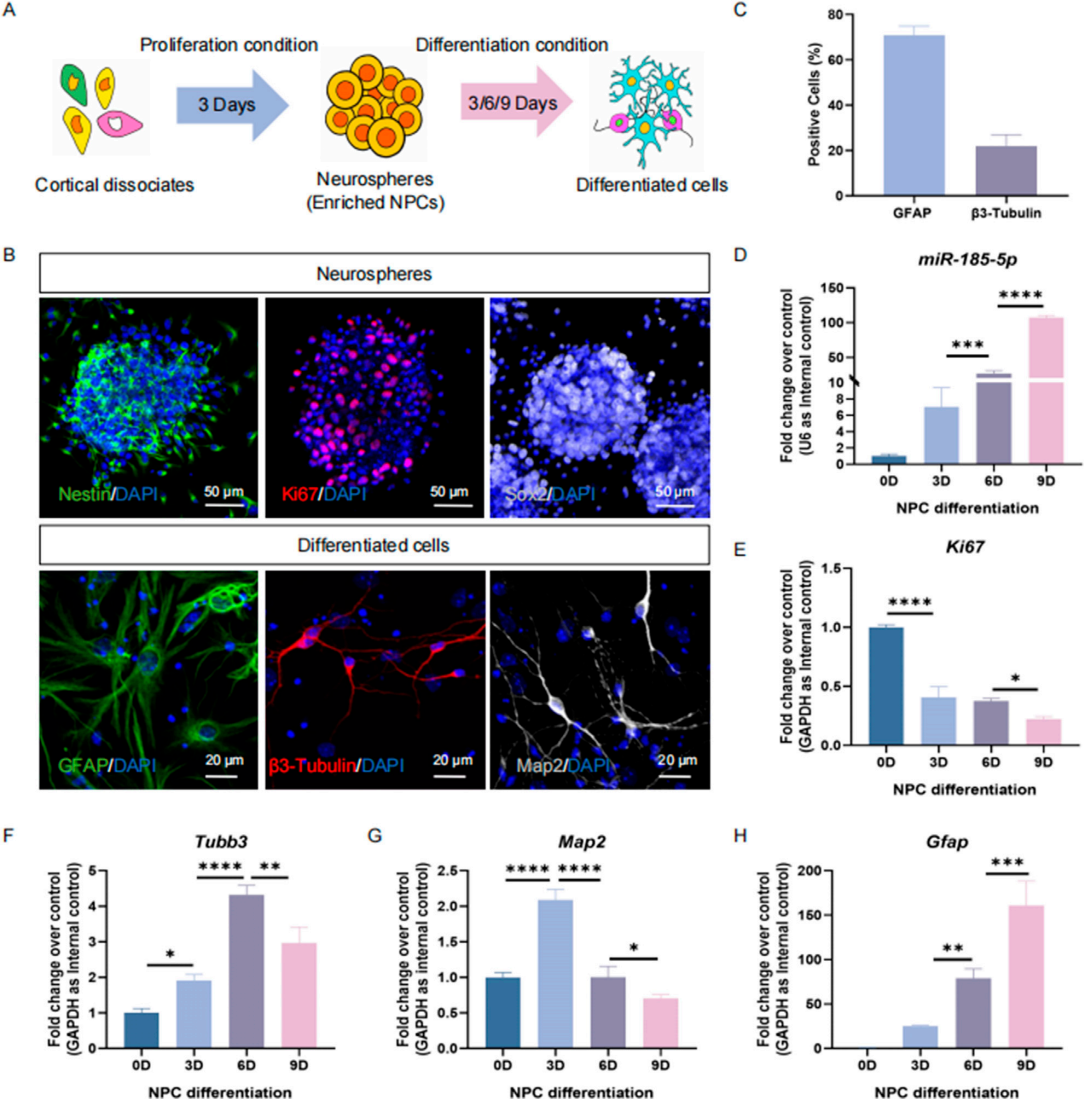
The temporal expression pattern of *miR-185-5p* corresponds to NPCs differentiation *in vitro*. **(A)** A schematic representation of the enrichment and differentiation of NPCs. **(B)** The enrichment and differentiation of NPCs was confirmed by immunoreactivities corresponding to protein markers of NPCs (β3-Tubulin-positive/Ki67/Sox2) and differentiated cells (GFAP/β3-Tubulin/Map2), respectively. Scale bar: 50 μm and 20 μm. **(C)** Number of GFAP-positive cells and β3-Tubulin-positive cells were quantified using Image J. **(D)** The expression levels of *miR-185-5p* during NPCs’ differentiation were determined by RT-qPCR analysis. **(E–H)** The expression levels of *Gfap/Tubb3/Map2/Ki67* transcripts during NPCs’ differentiation were determined by RT-qPCR analysis. Data are mean ± S.D. ∗∗∗∗p < 0.0001, ∗∗∗p < 0.001, ∗∗p < 0.01, and ∗p < 0.05. Experiments were carried out three times in triplicates.

Next, we determined the expression pattern of *miR-185-5p* in NPCs and differentiated cells *in vitro* (Figure 2A). E14 cortical dissociates cultured in the presence of EGF and FGF2 produced neurospheres enriched with Ki67/Nestin/Sox2 cells 3 days after inoculation, indicating an enrichment of NPCs (Figure 2B). NPCs were cultured under differentiation conditions and differentiated into β3-Tubulin-positive/Map2-positive neurons and GFAP^+^ glial cells (Figures 2B, C). The qPCR results showed that *miR-185-5p* was lowly expressed in NPCs but highly expressed in differentiated cells, and *miR-185-5p* expression gradually increased with the increase of differentiation days (Figure 2D), which had the same expression pattern as the transcript corresponding to *Gfap* and was different from *Ki67, Tubb3, and Map2* (Figures 2E–H). Thus, *in vivo* and *in vitro* studies demonstrated a corresponding positive correlation between *miR-185-5p* expression and the differentiation of NPCs, especially with astrocytes, suggesting that its function is involved in the regulation of NPCs differentiation.

### *miR-185-5p* negatively regulates the proliferation and self-renewal of NPCs

**FIGURE 3:**
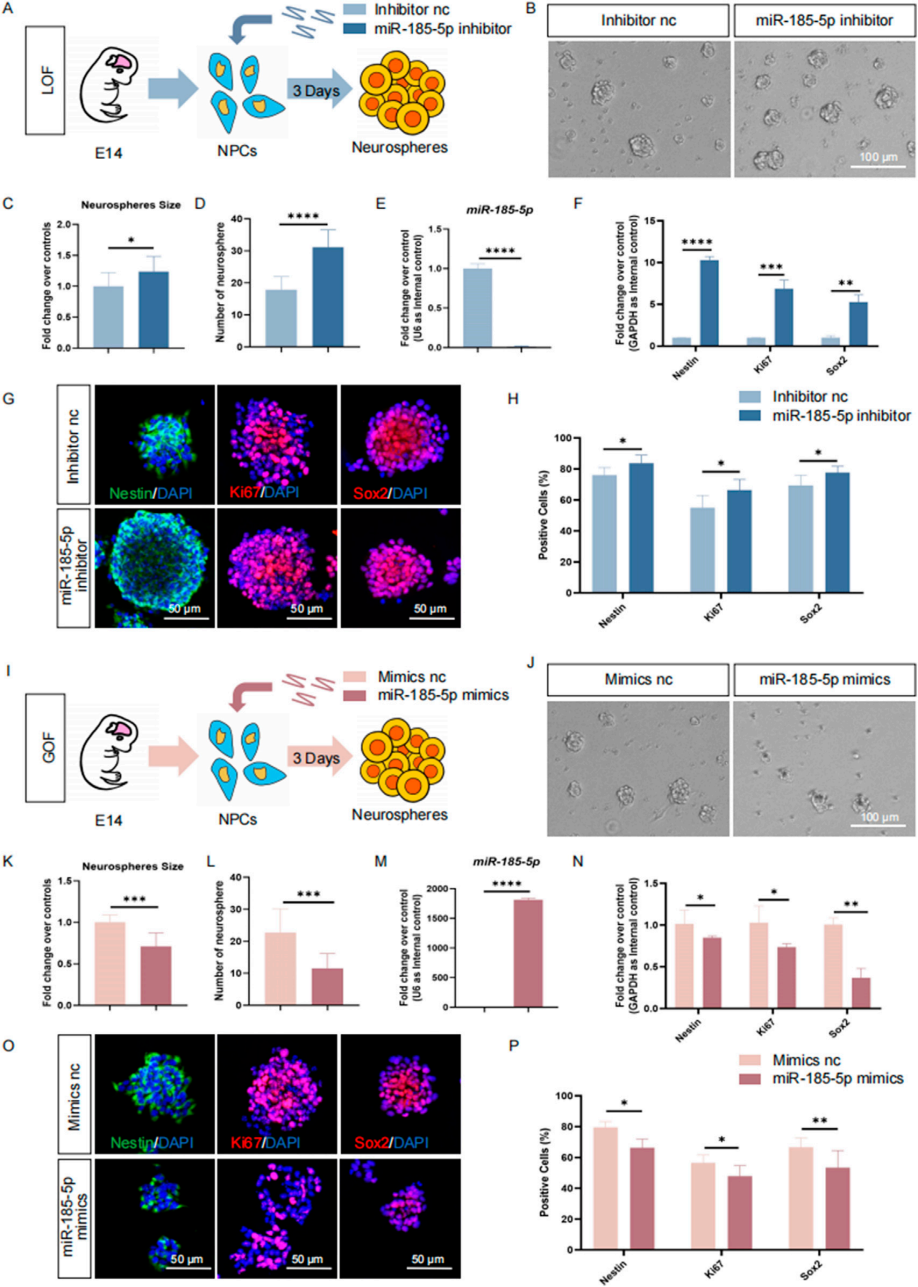
*miR-185-5p* inhibited the proliferation of NPCs. **(A)** A schematic representation of *miR-185-5p* LOF approach. **(B)** Bright field images of neurospheres in *miR-185-5p* LOF and control groups. **(C, D)** The number **(C)** and size **(D)** of neurospheres were quantified in the *miR-185-5p* LOF group, compared with controls. **(E, F)** RT-qPCR analysis of expression levels of *miR-185-5p* and transcripts corresponding to markers of NPCs (*Nestin/Sox2*) and proliferating cells (*Ki67*) in the *miR-185-5p* LOF groups, compared with controls. **(G)** Immunofluorescence analysis of transduced cells displaying NPCs (Nestin/Sox2) and proliferating cells (Ki67) specific immunoreactivities in LOF experiments. Scale bar: 50 μm. **(H)** Quantification of cells displaying immunoreactivities corresponding to NPCs and proliferating cells in the *miR-185-5p* LOF group, compared with controls. **(I)** A schematic representation of *miR-185-5p* GOF approach. **(J)** Bright field images of neurospheres in *miR-185-5p* LOF and control groups. **(K, L)** The number **(K)** and size **(L)** of neurospheres were quantified in the *miR-185-5p* GOF group, compared to controls. **(M, N)** RT-qPCR analysis of expression levels of *miR-185-5p* and transcripts corresponding to markers of NPCs (*Nestin/Sox2*) and proliferating cells (*Ki67*) in the *miR-185-5p* GOF groups, compared with controls. **(O)** Immunofluorescence analysis of transduced cells displaying NPCs (Nestin/Sox2) and proliferating cells (Ki67) specific immunoreactivities in GOF experiments. Scale bar: 50 μm. **(P)** Quantification of cells displaying immunoreactivities corresponding to NPCs and proliferating cells in the *miR-185-5p* GOF group, compared with controls. Data are mean ± S.D. ∗∗∗∗p < 0.0001, ∗∗∗p < 0.001, ∗∗p < 0.01, and ∗p < 0.05. Experiments were carried out three times in triplicates for *in vitro* perturbation.

To understand the roles of *miR-185-5p* in the regulation of NPCs, we first investigated the involvement of *miR-185-5p* in the proliferation of NPCs. *miR-185-5p* loss-of-function (LOF) and gain-of-function (GOF) assays were performed using *miR-185-5p*-specific inhibitors and mimics, respectively (Figures 3A, I). In the *miR-185-5p* LOF assay, NPCs were transfected with either *miR-185-5p* inhibitor or inhibitor negative control (nc), and cultured under proliferative conditions for 3 days (Figure 3A). After 3 days’ culture, a significant increase in the number and size of neurospheres (Figures 3B–D) was observed, indicating enhanced proliferation and self-renewal of NPCs in the *miR-185-5p* inhibitor group compared to the inhibitor nc group. Transfection efficiency was verified by RT-qPCR (Figure 3E). The results revealed significantly reduced *miR-185-5p* expression levels in the *miR-185-5p* inhibitor group, compared with controls (Figure 3E). RT-qPCR analysis further showed that the expression levels of transcripts corresponding to *Nestin*, *Sox2*, and *Ki67* were significantly increased in the *miR-185-5p* inhibitor group versus controls (Figure 3F). Moreover, the proportion of immunoreactive cells of Ki67, Nestin, and Sox2 in the *miR-185-5p* inhibitor group was significantly increased, compared with the inhibitor nc group (Figures 3G, H), confirming that *miR-185-5p* LOF promotes the proliferation of NPCs.

Next, we performed GOF using the same strategy as *miR-185-5p* LOF, in which NPCs were transfected with either *miR-185-5p* mimics or mimics negative control (nc) and cultured under proliferative conditions for 3 days (Figure 3I). We noticed a significant increase in the number and size of neurospheres in the group transfected with the *miR-185-5p* mimic, indicating enhanced proliferation and self-renewal of NPCs (Figures 3J–L). RT-qPCR analysis showed a significant increase in *miR-185-5p* levels (Figure 3M), validating the transfection efficiency. Compared with the mimics nc group, the *miR-185-5p* mimics group showed downregulation of *Nestin*, *Sox2* and *Ki67* transcript levels (Figure 3N), as well as a reduced proportion of cells with Ki67/Nestin/Sox2 specific immunoreactivity (Figures 3O, P), suggesting inhibition of proliferation and self-renewal of NPCs. Thus, our results suggest that *miR-185-5p* negatively regulates the proliferation of NPCs.

### *miR-185-5p* facilitates the differentiation of NPCs into astroglial lineage

**FIGURE 4:**
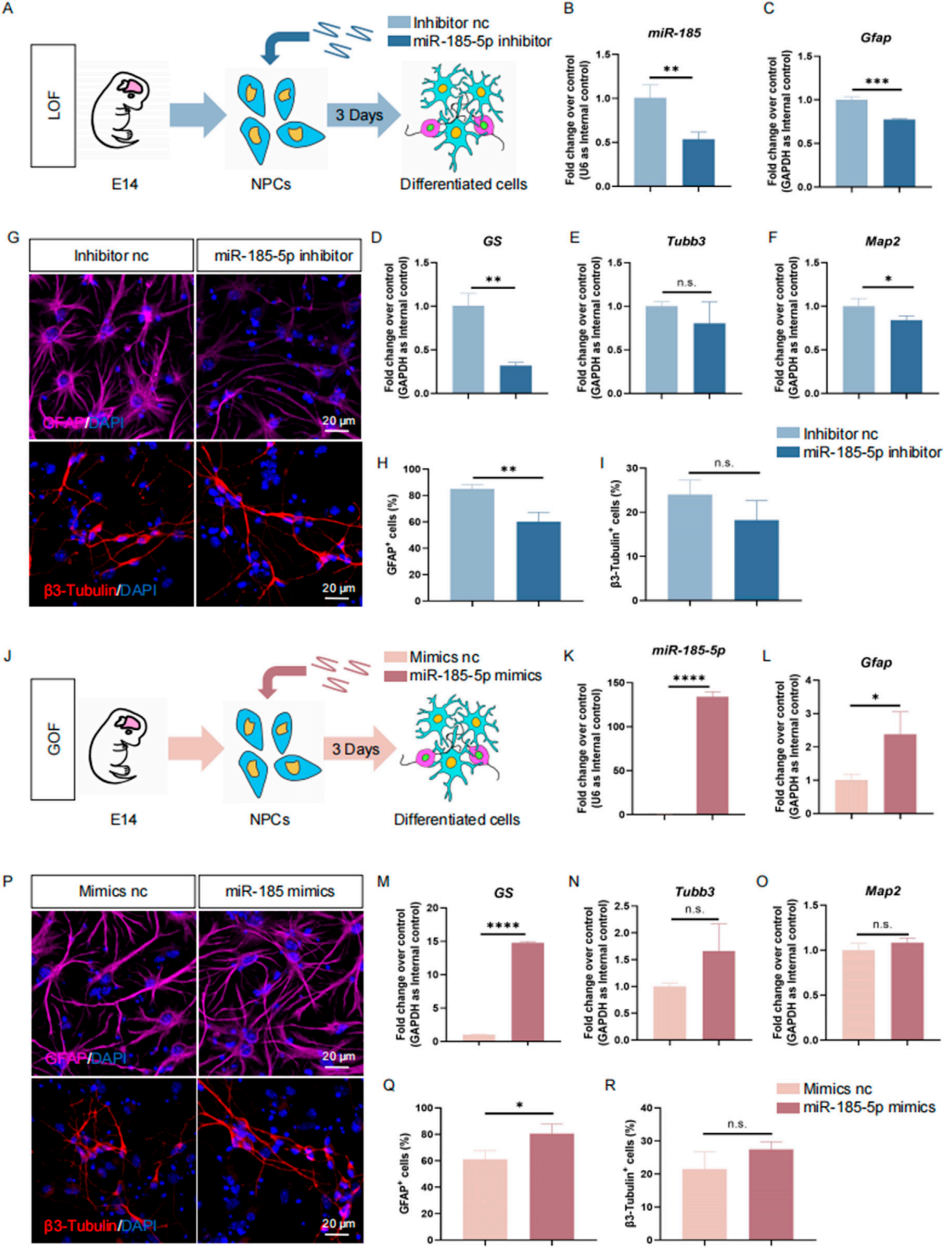
*miR-185-5p* promoted the differentiation of NPCs. **(A)** A schematic representation of *miR-185-5p* LOF approach. **(B–F)** RT-qPCR analysis of expression levels of *miR-185-5p* and transcripts corresponding to markers of differentiated cells (*Gfap/GS/Tubb3/Map2*) in the *miR-185-5p* LOF groups, compared with controls. **(G)** Representative images of immunofluorescence staining for the astrocyte-specific marker GFAP and the neuron-specific marker β3-Tubulin in LOF experiments. Scale bar: 20 μm. **(H)** Quantitative analysis of the number of GFAP-positive cells in LOF experiments. **(I)** Quantitative analysis of the number of β3-Tubulin-positive cells in LOF experiments. **(J)** A schematic representation of *miR-185-5p* GOF approach. **(K–O)** RT-qPCR analysis of expression levels of *miR-185-5p* and transcripts corresponding to markers of differentiated cells (*Gfap/GS/Tubb3/Map2*) in the *miR-185-5p* GOF groups, compared with controls. **(P)** Representative images of immunofluorescence staining for the astrocyte-specific marker GFAP and the neuron-specific marker β3-Tubulin in GOF experiments. Scale bar: 20 μm. **(Q)** Quantitative analysis of the number of GFAP-positive cells in GOF experiments. **(R)** Quantitative analysis of the number of β3-Tubulin-positive cells in GOF experiments. Data are mean ± S.D. ∗∗∗∗p < 0.0001, ∗∗∗p < 0.001, ∗∗p < 0.01, and ∗p < 0.05. Experiments were carried out three times in triplicates for *in vitro* perturbation.

Next, we investigated the role of *miR-185-5p* in the differentiation of NPCs. Similar to previous studies, we performed LOF and GOF approaches to explore the role of *miR-185-5p* under differentiation conditions. We transfected NPCs with *miR-185-5p* inhibitor or inhibitor nc and cultured them under differentiation conditions for 3 days (Figure 4A). RT-qPCR analysis showed a significant decrease in *miR-185-5p* levels (Figure 4B), validating the transfection efficiency. We observed a reduction in the transcript expression levels of the astrocyte-specific markers *Gfap* and *GS* (Figures 4C, D), and of the neuron-specific marker *Map2* compared to controls, with no significant difference in *Tubb3* (Figures 4E, F). Immunofluorescence analysis showed a significant reduction in the percentage of immunoreactivity for GFAP (Figures 4G, H) and the percentage of immunoreactivity for β3-Tubulin showed a decreasing trend but no significant difference (Figure 4I), confirming that *miR-185-5p* LOF inhibited the differentiation of NPCs, especially their differentiation to astrocytes. In *miR-185-5p* GOF analysis, NPCs were transfected with *miR-185-5p* mimics or negative controls and cultured for 3 days under differentiation conditions (Figures 4J, K). Ectopic expression of *miR-185-5p* significantly increased the differentiation of glial cells compared to the results obtained by the LOF method, which was confirmed by a significant increase in the transcript levels corresponding to cell type-specific markers (Figures 4L–O) and in the number of cells displaying cell type-specific immunoreactivity (Figures 4P–R). Thus, both LOF and GOF studies suggest that *miR-185-5p* positively regulates the differentiation of NPCs, especially their differentiation to astrocytes.

**FIGURE 5:**
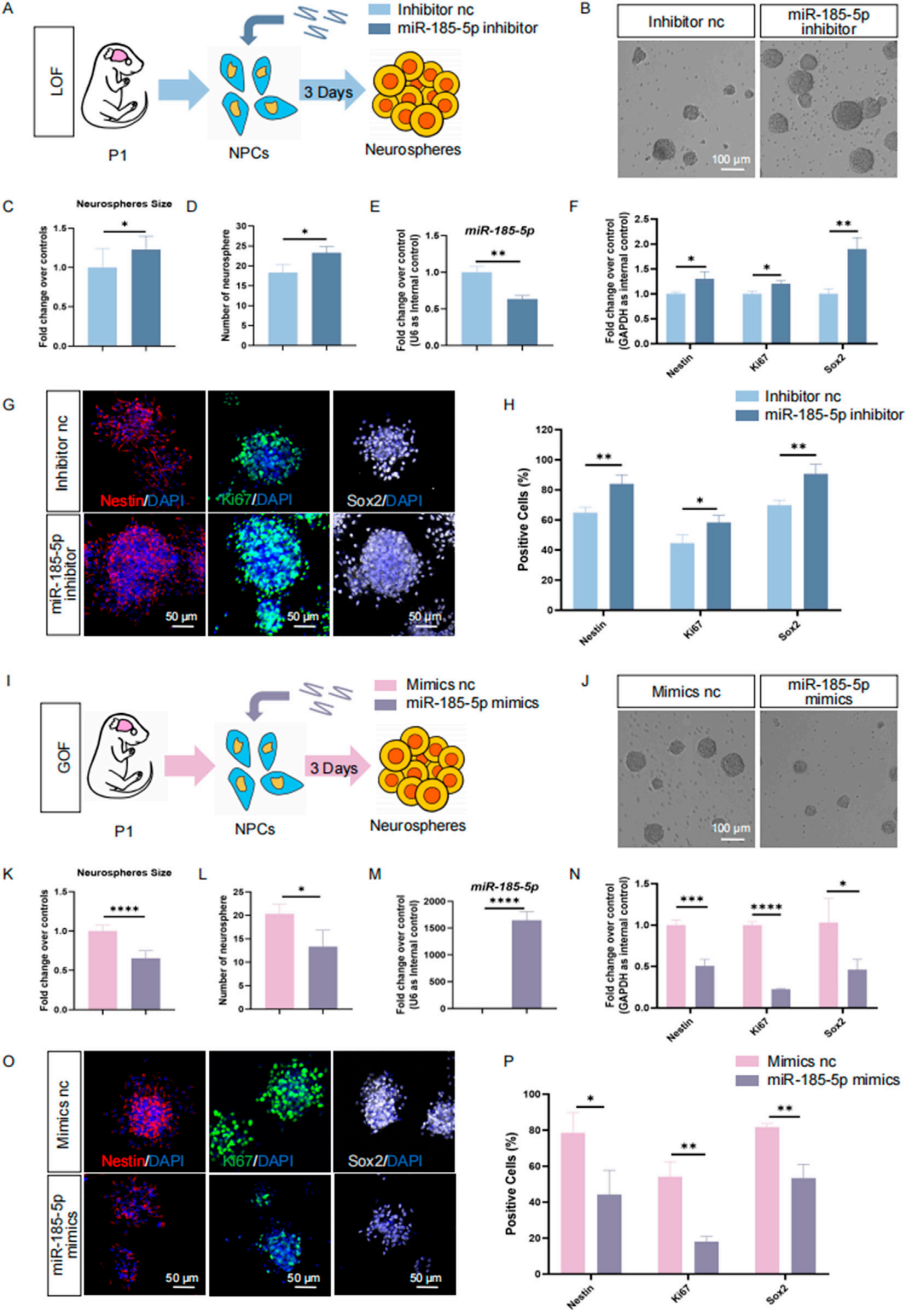
*miR-185-5p* inhibits proliferation of P1 NPCs. **(A)** A schematic representation of *miR-185-5p* LOF approach. **(B)** Bright field images of neurospheres in *miR-185-5p* GOF and control groups. **(C, D)** The number **(C)** and size **(D)** of neurospheres were quantified in the *miR-185-5p* LOF group, compared with controls. **(E, F)** RT-qPCR analysis of expression levels of *miR-185-5p* and transcripts corresponding to markers of NPCs (*Nestin/Sox2*) and proliferating cells (*Ki67*) in the *miR-185-5p* LOF groups, compared with controls. **(G)** Immunofluorescence analysis of transduced cells displaying NPCs (Nestin/Sox2) and proliferating cells (Ki67) specific immunoreactivities in LOF experiments. Scale bar: 50 μm. **(H)** Quantification of cells displaying immunoreactivities corresponding to NPCs and proliferating cells in the *miR-185-5p* LOF group, compared with controls. **(I)** A schematic representation of *miR-185-5p* GOF approach. **(J)** Bright field images of neurospheres in *miR-185-5p* LOF and control groups. **(K, L)** The number **(K)** and size **(L)** of neurospheres were quantified in the *miR-185-5p* GOF group, compared with controls. **(M, N)** RT-qPCR analysis of expression levels of *miR-185-5p* and transcripts corresponding to markers of NPCs (*Nestin/Sox2*) and proliferating cells (*Ki67*) in the *miR-185-5p* GOF groups, compared with controls. **(O)** Immunofluorescence analysis of transduced cells displaying NPCs (Nestin/Sox2) and proliferating cells (Ki67) specific immunoreactivities in GOF experiments. Scale bar: 50 μm. **(P)** Quantification of cells displaying immunoreactivities corresponding to NPCs and proliferating cells in the *miR-185-5p* GOF group, compared with controls. Data are mean ± S.D. ∗∗∗∗p < 0.0001, ∗∗∗p < 0.001, ∗∗p < 0.01, and ∗p < 0.05. Experiments were carried out three times in triplicates for *in vitro* perturbation.

**FIGURE 6:**
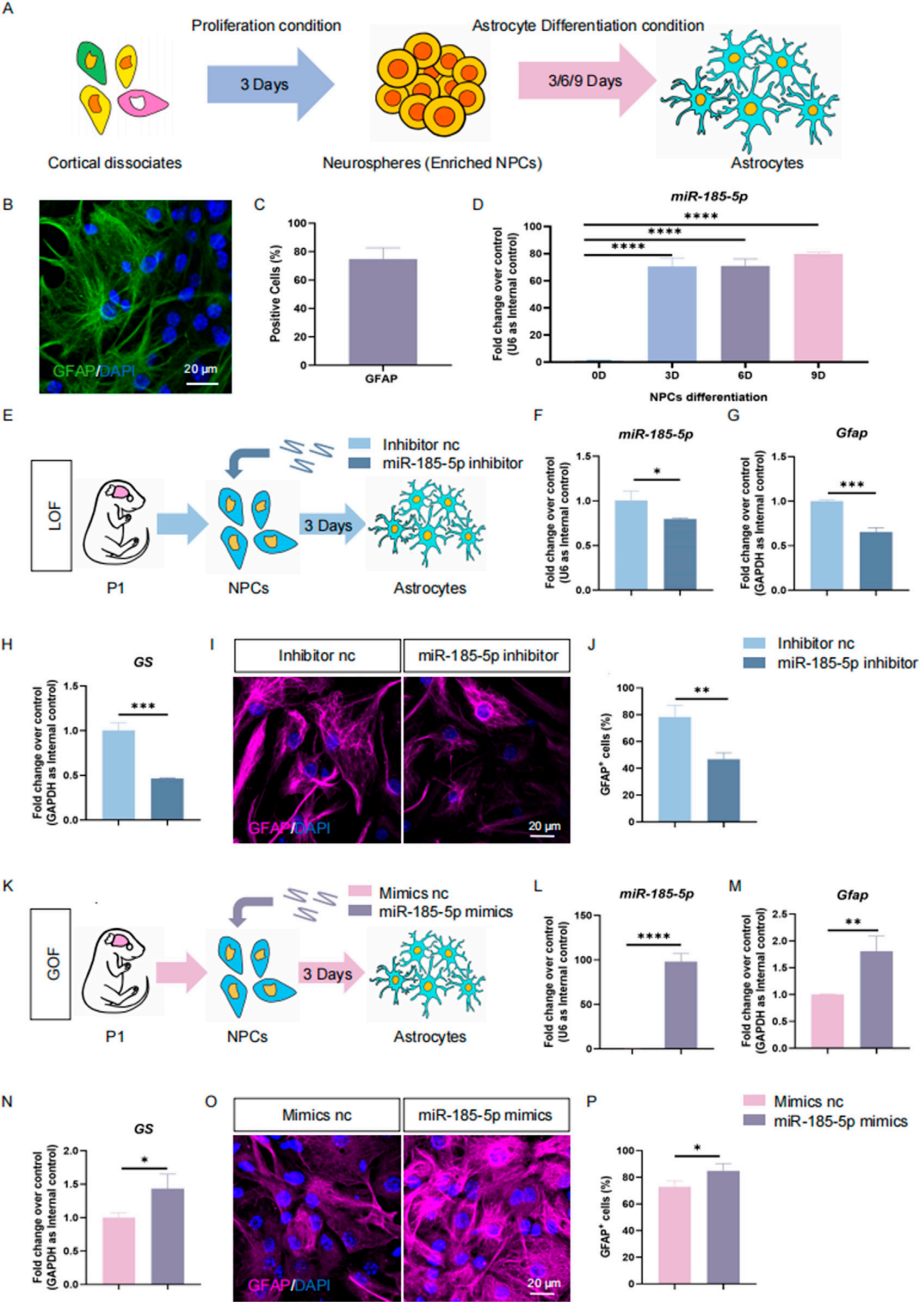
*miR-185-5p* promotes directed differentiation of P1 NPCs. **(A)** A schematic representation of the enrichment and astrocytes differentiation of NPCs. **(B)** Number of GFAP-positive cells were quantified using Image J. **(C)** Immunofluorescence staining for the astrocyte-specific marker GFAP identifies differentiation of NPCs to astrocytes. Scale bar: 20 μm. **(D)** RT-qPCR analysis showed that the expression levels of *miR-185-5p* were elevated in the differentiation of NPCs to astrocytes. **(E)** A schematic representation of *miR-185-5p* LOF approach. **(F–H)** RT-qPCR analysis of expression levels of *miR-185-5p* and transcripts corresponding to markers of astrocytes (*Gfap/GS*) in the *miR-185-5p* LOF groups, compared with controls. **(I)** Representative images of immunofluorescence staining for the astrocyte-specific marker GFAP in LOF experiments. Scale bar: 20 μm. **(J)** Quantitative analysis of the number of GFAP-positive cells in LOF experiments. **(K)** A schematic representation of *miR-185-5p* GOF approach. **(L–N)** RT-qPCR analysis of expression levels of *miR-185-5p* and transcripts corresponding to markers of astrocytes (*Gfap/GS*) in the *miR-185-5p* GOF groups, compared with controls. **(O)** Representative images of immunofluorescence staining for the astrocyte-specific marker GFAP in GOF experiments. Scale bar: 20 μm. **(P)** Quantitative analysis of the number of GFAP-positive cells in GOF experiments. Data are mean ± S.D. ∗∗∗∗p < 0.0001, ∗∗∗p < 0.001, ∗∗p < 0.01, and ∗p < 0.05. Experiments were carried out three times in triplicates for *in vitro* perturbation.

To further confirm the positive effects of *miR-185-5p* on NPCs’ differentiation to astrocyte, we isolated mouse NPCs in P1, a stage when gliogenesis occupies a dominant position versus neurogenesis (Dwyer et al., 2016; Guerra, 2014). We also performed proliferation experiments in P1 mouse-derived NPCs, and the results of our LOF assay (Figures 5A–H) and GOF assay (Figures 5I–P) indicated that *miR-185-5p* inhibited the proliferation and self-renewal of NPCs, which is consistent with the results we obtained in E14-derived NPCs. We induced these P1 NPCs to differentiate into astrocytes by culturing them in astrocyte differentiation medium for 3 days (Figures 6A–C). RT-qPCR analysis detected a significant increase in *miR-185-5p* expression after differentiation of NPCs to astrocytes (Figure 6D). Afterwards, both *miR-185-5p* LOF and GOF were conducted on P1 NPCs to determine the effects of *miR-185-5p* on astrocyte differentiation (Figures 6E, K). In the LOF assay, we verified the transfection efficiency of the *miR-185-5p* inhibitor (Figure 6F) and found that the mRNA levels of *Gfap* and *GS* were reduced in the *miR-185-5p* inhibitor group (Figures 6G, H) and the number of GFAP-positive cells was decreased in the immunofluorescence analysis compared to the inhibitor nc group (Figures 6I, J). In the GOF assay, we similarly verified the transfection efficiency of the *miR-185-5p* mimics (Figure 6L) and found increased mRNA levels of *Gfap* and *GS* in the *miR-185-5p* mimics group compared to the mimics nc group (Figures 6M, N), as verified by an increase in the number of GFAP-positive cells by immunofluorescence analysis (Figures 6O, P). The above results suggest that *miR-185-5p* is capable of promoting the differentiation of NPCs to astrocytes.

### *miR-185-5p* regulates proliferation and differentiation of NPCs *in vivo*

**FIGURE 7:**
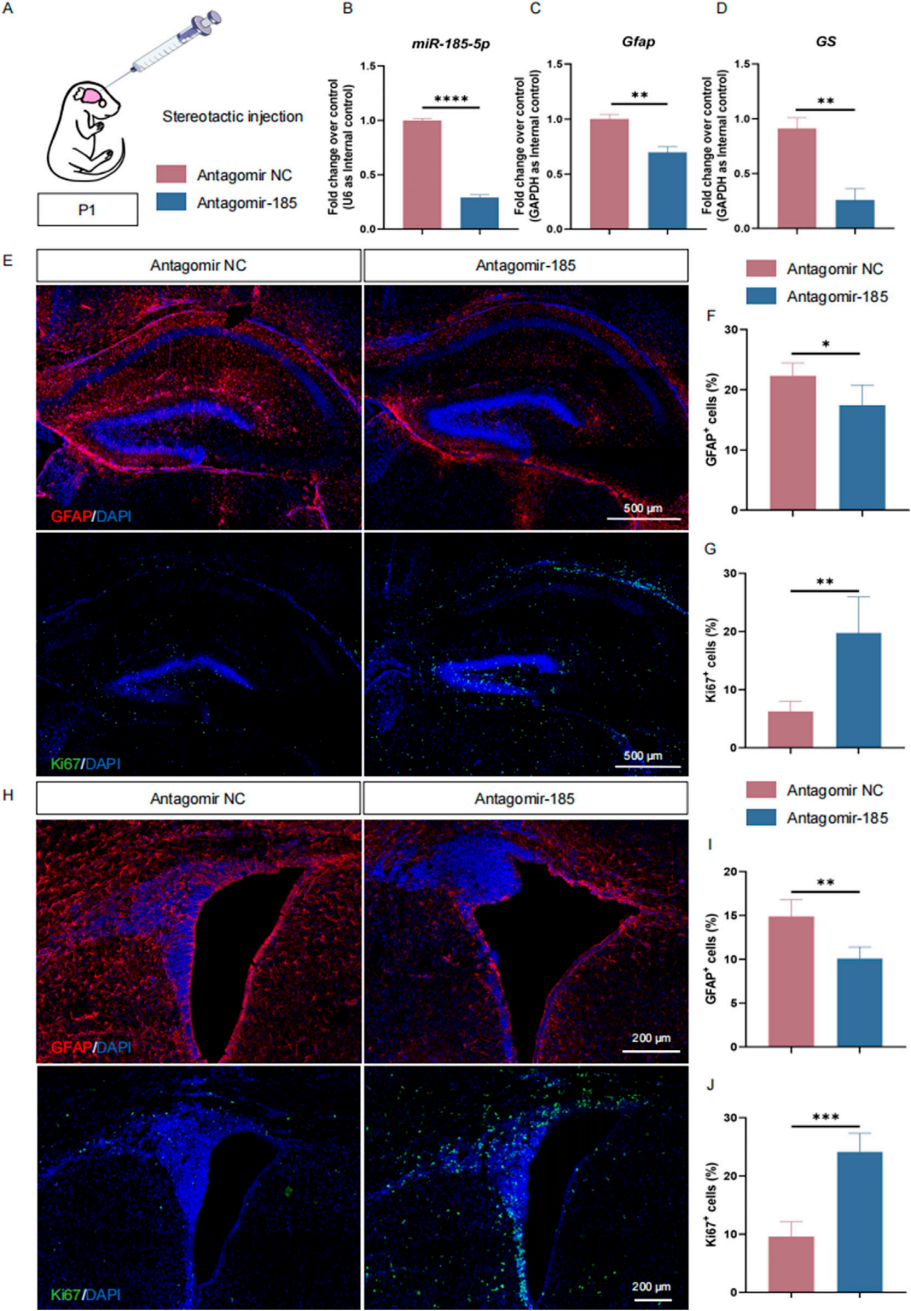
*miR-185-5p* regulates the proliferation and differentiation of NPCs *in vivo*. **(A)** A schematic representation of *miR-185-5p* LOF approach *in vivo*. **(B–D)** RT-qPCR analysis of expression levels of *miR-185-5p* and transcripts corresponding to markers of astrocytes (*Gfap/GS*) in the *miR-185-5p* LOF groups, compared with controls (*n* = 3). **(E–G)** Immunofluorescence analysis and quantification of transduced cells displaying GFAP and Ki67 specific immunoreactivities in the SGZ (*n* = 3). **(H–J)** Immunofluorescence analysis and quantification of transduced cells displaying GFAP and Ki67 specific immunoreactivities in the SVZ (*n* = 3). Scale bar: 500 μm and 200 μm. Data are mean ± S.D. ∗∗∗∗p < 0.0001, ∗∗∗p < 0.001, ∗∗p < 0.01, and ∗p < 0.05. Experiments were carried out three times in triplicates for *in vivo* perturbation.

Our *in vitro* studies demonstrated the critical importance of *miR-185-5p* in promoting the differentiation of NPCs, especially astrocytes. To further validate our observations, we performed *miR-185-5p* knockdown in P1 C57BL/6J mice. The lateral ventricles of P1 mice were injected with antagomir-185–5p (=LOF group) or antagomir negative control (NC), and the animals were executed 72 h later (Figure 7A). The expression of *miR-185-5p* was suppressed in the brain tissues of mice in the *miR-185-5p* LOF group compared with the antagomir NC group (Figure 7B), indicating that *miR-185-5p* was successfully knocked down *in vivo*. RT-qPCR results showed that the expression of transcripts corresponding to *Gfap* and *GS* was significantly reduced in the *miR-185-5p* LOF group compared with the antagomir NC group (Figures 7C, D). The proportion of cells showing GFAP immunoreactivity was significantly decreased in the *miR-185-5p* LOF group compared to the antagomir NC group in both the subgranular zone (SGZ) of the hippocampal dentate gyrus (Figures 7E, F) and the subventricular zone (SVZ) of the lateral ventricles, two main regions where NPCs exist in the mammalian brain throughout adulthood (Figures 7H, I). In addition, *miR-185-5p* LOF enhanced the proliferation of NPCs compared to the antagomir NC group, which was determined by the increase in the number of Ki67-positive cells in the *miR-185-5p* LOF group in the SGZ (Figures 7E,G) and SVZ (Figures 7H, J). Thus, our results suggest that *miR-185-5p* plays an essential role in promoting the differentiation of NPCs *in vivo*.

## Discussion

The development of the vertebrate CNS is a highly conserved and dynamic process, characterized by distinct stages that begin with the formation of the neural tube (Rakic, 2009; Jessell and Sanes, 2000). The cell fate commitment of NPCs, including self-renewal and differentiation into diverse lineages, is crucial throughout these stages (Thor, 2024; Yoon et al., 2018). Deregulation of NPCs during CNS development leads to significant developmental abnormalities, including malformations and intellectual disabilities (Jessell and Sanes, 2000). Identifying the critical factors involved in the regulation of NPCs is essential for understanding CNS development, and could be of great advantage to the prevention and treatment of developmental defects.

In recent years, miRNAs have been found to regulate both the proliferation and differentiation capacities of NPCs *in vitro* and *in vivo*. In this study, we found that the expression levels of *miR-185-5p* increased as neurodevelopment proceeds in mouse cortical and hippocampal tissues. We also extracted primary NPCs to confirm the positive correlation of the expression levels of *miR-185-5p* with NPCs differentiation, and the negative correlation with NPCs proliferation. We then implemented LOF and GOF approaches, and found that *miR-185-5p* inhibits the proliferation of E14 NPCs and promotes the differentiation of NPCs into astrocytes *in vitro*. These effects of *miR-185-5p* on NPCs were further validated by perturbing the expression of *miR-185-5p* in NPCs isolated from mouse brains at P1, the time point when *miR-185-5p* expression reaches the maximum levels. More importantly, the lateral ventricle administration of antagomir-185-5p repressed the generation of astrocytes and enhanced cell proliferation in the SVZ and SGZ, two key brain regions where NPCs reside in the adulthood (Doetsch et al., 1997), matching with our observations *in vitro*.

Prior to our study, multiple studies of *miR-185-5p* have shown that *miR-185-5p* suppress tumor cell division, suggesting *miR-185-5p* an anti-tumor (anti-proliferation) miRNA (Wang et al., 2021; Tang et al., 2014). Afterwards, studies revealed that *miR-185-5p* also functions as an anti-proliferation miRNAs in many types of normal cells, including endothelial cells (Feng et al., 2022), aortic vascular smooth muscle cells (Wang et al., 2017), and mesangial cells (Wang et al., 2017). In our study, *miR-185-5p* significantly suppresses the proliferation of NPCs derived from embryonic and postnatal mouse brains. Thus, our results, together with others’ findings, indicate that *miR-185-5p* as a general blocker of proliferation not only in the CNS, but also in other types of cells under physiological and pathological conditions. Notably, the mechanisms of *miR-185-5p* -mediated proliferation inhibition of NPCs remains unclear. Inspiringly, researches on other types of cells can provide us with many insights. For example, *miR-185-5p* inhibit the proliferation of tumor cells by targeting multiple genes, including *ROCK2* (Niu and Tang, 2019), *IGF2* (Zhuang et al., 2020), *RAGE* (Yin et al., 2018), *HMGA2* (Lu et al., 2022), *BCL2* (Değerli et al., 2020), *BCL2L1* (Değerli et al., 2020; Ostadrahimi et al., 2018), and *KLF7* (Zhao et al., 2019). Our previous study has identified HMGA2, a key chromatin-associated protein, as a key gene enhancing proliferation of RPCs (Xia and Ahmad, 2016; Parameswaran et al., 2014). Besides, the inhibition of either ROCK (Zhang et al., 2016), IGF2 (Yang et al., 2015), or RAGE (Li et al., 2014) pathways has also been reported to suppress the proliferation of NSCs. Hence, aforementioned may regulate proliferation of NPCs *via* inhibiting aforementioned pathways, which needs to be investigated in the future studies.

Besides the anti-proliferation effect, we also found that *miR-185-5p* plays a key role in gliogenesis *in vitro* and *in vivo*. The development of the CNS follows a strict sequence, and one of the well-established aspects is that neurogenesis precedes gliogenesis. There are multiple miRNAs that are found to regulate neurogliogenesis decision including *miR-9*, *miR-124*, and *miR-29* (Xia et al., 2019b; Åkerblom and Jakobsson, 2014; Coolen et al., 2013). For example, we have found the *miR-9* enhances neurogenesis and inhibits gliogenesis by targeting *HES1*, a downstream transcription factor of NOTCH signaling (Yuan et al., 2021). Moreover, *miR-9* also enhances neurogenesis through *MCPIP1* (Yang et al., 2013), *ELAVL3* (Coolen et al., 2012), *TLX* (Madelaine et al., 2017), and *ONECUTs* (Madelaine et al., 2017). Another well-recognized pro-neural miRNA is *miR-124*, which promotes neurogenesis *via* targeting *NEUROD1* (Liu et al., 2011) and *AAK1* (Song et al., 2023). Besides, our recent study also identified *miR-29* as a regulator of neuroligogenesis by down-regulating *REST* expression (Xia et al., 2019b). Here, we identified *miR-185-5p* as a novel miRNA for promoting gliogenesis, although the underlying molecular mechanisms remains unknown. Based on the information from online database Targetscan.org, *miR-185-5p* does not directly binds to the well-known *miR-9*/*124*/*29* target transcripts like *HES1*, *TLX*, and *REST*, suggesting that *miR-185-5p* regulates NPCs proliferation and differentiation through distinct mechanisms. Interestingly, in addition to regulating proliferation, HMGA2 also has the functions of enhancing neurogenesis and suppressing gliogenesis through regulating HES5, a NOTCH effector and well-established node that facilitates the astrocytic fate commitment in the CNS (Bansod et al., 2017; Patterson et al., 2014). These findings imply *HMGA2* as a core downstream target of *miR-185-5p* that mediates the anti-proliferative and pro-glial effects of *miR-185-5p* on NPCs. Another possible target of *miR-185-5p* in the regulation of gliogenesis is IGF2 pathway (Zhuang et al., 2020), which controls the timing of neurogliogenesis during brain development (Balzer et al., 2010). Future investigations that aim to clarify the molecular mechanisms of *miR-185-5p*-mediated astrocytic commitment are urgently needed.

It is worth noting that, besides the regulatory effects on NPCs and brain development, *miR-185-5p* also participate in the modulation of neurological disease progression. For example, we detected reduced *miR-185-5p* levels in the serum of Alzheimer’s disease (AD) patients (Ding et al., 2022). We further found that *miR-185-5p* negatively regulates the expression of amyloid precursor protein (APP) through the direct binding to the APP 3′UTR (Ding et al., 2022). Therefore, exosomal *miR-185-5p* in the microenvironment of the CNS functions as a blocker for APP expression, and the loss of *miR-185-5p* in exosomes releases *APP* mRNAs from degradation or translational inhibition in the recipient cells to promote Aβ production (Ding et al., 2022). Downregulation of *miR-185-5p* has also been identified as a common pathogenic event in 22q11.2 deletion syndrome-related and idiopathic schizophrenia (Sabaie et al., 2022). Moreover, reduced *miR-185-5p* levels were found in glioma tissues, indicating that *miR-185-5p* was associated with a poor outcome in glioma patients (Tang et al., 2012). Thus, these observations, together with our findings, imply a great potential of *miR-185-5p* as a biomarker for neurological diseases, particularly development disorders and brain tumors that directly associate with dysregulated proliferation of normal or cancer cells in the brains. For instance, lowered circulating *miR-185-5p* has been reported as a predictive biomarker for diagnosis and prognosis of glioma (Tabibkhooei et al., 2020; Tang et al., 2015). Besides, as anti-proliferative effects of *miR-185-5p* have been reported in brain tumor (Guan et al., 2022), *miR-185-5p* may also serve as a potential drug for brain tumors, which will be investigated in our future works.

In conclusion, we found that *miR-185-5p* expression levels increase during both the development of mouse brains and differentiation process of NPCs. The upregulation of *miR-185-5p* expression suppresses proliferation of NPCs and facilitates astrocyte differentiation *in vitro* and *in vivo*. Thus, our study identified *miR-185-5p* as a novel player in the cell fate commitment of NPCs, suggesting the complexity of miRNA-mediated regulation in the brain development.

## Data Availability

The original contributions presented in the study are included in the article/Supplementary Material, further inquiries can be directed to the corresponding authors.

## Abbreviations

NPCs, neural stem/progenitor cells; miRNA, microRNA; 3′UTR, 3′ untranslated region; CNS, central nervous system; E14, embryonic day 14; E18, embryonic day 18; P1, postnatal day 1; SD, standard deviation; APP, amyloid precursor protein; AD, Alzheimer’s disease.
