# Supplementary material for: miR-185-5p regulates the proliferation and differentiation of neural stem/progenitor cells
Source: Front Cell Dev Biol. 2024 Dec 5;12:1510746. doi: 10.3389/fcell.2024.1510746 (PMC11656079; doi:10.3389/fcell.2024.1510746)
Supplement: Supplementary file 1 [file Table1.docx]

| Gene Symbol | Forward Primer | Reverse Primer |
| --- | --- | --- |
| *Gapdh* | CATGTTCCAGTATGACTCCACTC | GGCCTCACCCCATTTGATGT |
| *Gfap* | TTGCTGGAGGGCGAAGAAAA | CATCCCGCATCTCCACAGTC |
| *GS* | TCACAGGGACAAATGCCGAG | GTTGATGTTGGAGGTTTCGTGG |
| *Ki67* | CAGCAGAAGAATCGTGGGAGAC | CCTACTTTGGGTGAAGAGGTTGC |
| *Nestin* | GGAGAGTCGCTTAGAGGTGC | TCAGGAAAGCCAAGAGAAGC |
| *Sox2* | CCCAGCGCCCGCATGTATAA | GCGCTTGCTGATCTCCGAGT |
| *Tubb3* | TGGGCTCCCACAGTGGCTAT | GTGCCTCTGGCCCCATCATC |
| *Map2* | AGCCGCAACGCCAATGGATT | TTTGTTCTGAGGCTGGCGAT |
| *Universal primer* | GAATCGAGCACCAGTTACGC | |
| *U6* | TGGCCCCTGCGCAAGGATG | |
| *miR-185-5p* | TGGAGAGAAAGGCAGTTCCTGA | |

**Supplementary Table S1.**

| Antibodies | Source | Identifier |
| --- | --- | --- |
| GFAP | Millipore | AB35541 |
| Ki67 | Cell Signaling Technology | 9129 |
| Nestin | Cell Signaling Technology | 89529 |
| Sox2 | Cell Signaling Technology | 23064 |
| β3-Tubulin | Cell Signaling Technology | 5568 |
| Map2 | Cell Signaling Technology | 4542 |

**Supplementary Table S2.**
